# Erhöhtes Risiko für tiefe Beinvenenthrombosen bei Intensivpatienten mit CoViD-19-Infektion? – Erste Daten

**DOI:** 10.1007/s00104-020-01222-7

**Published:** 2020-06-05

**Authors:** Sebastian Zerwes, F. Hernandez Cancino, D. Liebetrau, Y. Gosslau, T. Warm, B. Märkl, A. Hyhlik-Dürr

**Affiliations:** 1 Gefäßchirurgie und endovaskuläre Chirurgie, Medizinische Fakultät, Universität Augsburg, Stenglinstraße 2, 86156 Augsburg, Deutschland; 2 Allgemeine und Spezielle Pathologie, Medizinische Fakultät, Universität Augsburg, Augsburg, Deutschland

**Keywords:** CoViD-19, Thrombose, Corona-Infektion, CoViD-19-assoziierte Komplikationen, Tiefe Beinvenenthrombose, CoViD-19, Thrombosis, Corona infection, CoViD-19 associated complications, Deep vein thrombosis

## Abstract

**Hintergrund:**

Die Inzidenz tiefer Beinvenenthrombosen (TVT) bei intensivpflichtigen CoViD-19-Patienten wurde bisher nur in wenigen Studien untersucht. Prospektive vergleichende Studien mit Non-CoViD-19-Intensivpatienten fehlen gänzlich.

**Fragestellung:**

Die Inzidenz TVT bei an CoViD-19 erkrankten Intensivpatienten verglichen mit Non-CoViD-19-Patienten, die im selben Zeitraum auf den Intensivstationen des Universitätsklinikums Augsburg behandelt wurden, wurden erhoben. Zudem soll untersucht werden, welche Art der Antikoagulation zum Zeitpunkt des Auftretens der TVT bei CoViD-19-Patienten vorlag und inwiefern eine TVT bei diesem Patientengut mit einer erhöhten Letalität vergesellschaftet ist.

**Material und Methoden:**

In der prospektiven Single-Center Studie wurden im Zeitraum vom 18.04.2020 bis 30.04.2020 20 SARS-CoV2-positive Patienten mit 20 Non-CoViD-Patienten auf Intensivstation bezüglich des Auftretens tiefer Beinvenenthrombosen verglichen. Hierzu wurden demographische Daten, Laborparameter und klinische Verläufe erfasst und ausgewertet.

**Ergebnisse:**

Die Rate an TVT war im untersuchten Kollektiv bei Patienten mit SARS-CoV2 deutlich erhöht (CoViD-19-Patienten: 20 % vs. Non-CoViD-19-Patienten: 5 %). Sowohl das Vorliegen einer TVT sowie deutlich erhöhte D‑Dimer-Werte waren in der vorliegenden Studie mit erhöhter Letalität vergesellschaftet.

**Diskussion:**

Wir empfehlen bei der stationären Aufnahme von Patienten mit SARS-CoV2-Verdacht oder Nachweis die Bestimmung der D‑Dimere und im Falle erhöhter Werte die großzügige Indikationsstellung zur Kompressionssonographie der tiefen Beinvenen. So können TVT früh erkannt und eine therapeutische Antikoagulation begonnen werden. Alle stationären CoViD-19-Patienten sollten eine Thromboseprophylaxe mit NMH erhalten. Weitere Studien zu Point-of-care-Methoden (TEG®, ROTEM®) zur Erkennung einer Hyperkoagulabilität bei SARS-CoV2 sind notwendig.

## Abstract (en)

**Background:**

The incidence of deep vein thrombosis (DVT) in CoViD-19 patients in intensive care units (ICU) has so far been investigated in only a few studies. Prospective comparative studies with non-CoViD-19 ICU patients are completely lacking.

**Objective:**

Evaluation of the incidence of DVT in ICU patients with CoViD-19 compared to non-CoViD-19 ICU patients who were treated in the University Hospital Augsburg during the same period. In addition, the aim was to investigate what type of anticoagulation was present in CoViD-19 patients at the time the DVT occurred and to what extent DVT is associated with increased mortality in this patient population.

**Material and methods:**

In this prospective single center study, which was conducted between 18 April 2020 and 30 April 2020, 20 SARS-CoV2 positive patients were compared with 20 non-CoVid-19 patients in the ICU with respect to the occurrence of DVT. For this purpose, demographic data, laboratory parameters, and clinical outcomes were recorded and evaluated.

**Results:**

The rate of DVT in the investigated patient collective was markedly higher in patients with SARS-CoV2 (CoViD-19 patients 20% vs. non-CoViD-19 patients 5%). Both DVT and elevated D‑dimer levels were associated with increased mortality in the present study.

**Conclusion:**

We recommend the determination of D‑dimer levels and, in the case of elevated levels, the broad indication for compression sonography of the deep leg veins on admission of patients with suspected or confirmed SARS-CoV2. In this way DVT in the setting of CoViD-19 can be recognized early and therapeutic anticoagulation can be started. All inpatient CoViD-19 patients should receive thrombosis prophylaxis with low molecular weight heparin. Further studies on point of care methods (TEG®, ROTEM®) for the detection of hypercoagulability in SARS-CoV2 are necessary.

## Hintergrund

Seit ihrem Ausbruch im Dezember 2019 in Wuhan, China, hat sich die CoViD-19-Infektion in rasanter Geschwindigkeit ausgebreitet und ist zu einer globalen Pandemie geworden [1]. Dabei scheint die CoViD-19-Erkrankung unter anderem das kardiovaskuläre System anzugreifen, was die aktuell beobachtete erhöhte Letalität begründen könnte [2–4]. Besonders die durch das SARS-CoV2-Virus mutmaßlich getriggerten Veränderungen der Gefäßinnenwand (Intima) durch eine ausgeprägte Inflammation sowie eine generelle Koagulopathie könnten die in mehreren aktuellen Studien belegte erhöhte Anzahl thrombembolischer Ereignisse im venösen und arteriellen System erklären [1, 5–7].

Es ist bekannt, dass intensivpflichtige Patienten per se ein erhöhtes Risiko für thrombembolische Ereignisse aufweisen: So treten bei Intensivpatienten Thrombosen je nach Grunderkrankung mit einer Wahrscheinlichkeit von 10–80 % auf; 10–15 % der Patienten entwickeln trotz Thromboseprophylaxe eine tiefe Beinvenenthrombosen (TVT; [8, 9]). Gründe hierfür können unter anderem Immobilisation, Veränderungen der Hämodynamik sowie Veränderungen der Blutzusammensetzung sein [10–12].

Wie hoch die Inzidenz tiefer Beinvenenthrombosen bei intensivpflichtigen CoViD-19-Patienten ist, wurde bisher jedoch nur in wenigen Studien untersucht; die angegebenen Raten variierten deutlich von 1–56 % [1, 5, 7]. Prospektive vergleichende Studien mit Non-CoViD-19-Intensivpatienten fehlen gänzlich.

## Fragestellung

Wir berichten in dieser prospektiven Single-Center-Untersuchung über die Inzidenz tiefer Beinvenenthrombosen bei 20 an CoViD-19 erkrankten Intensivpatienten, verglichen mit 20 Non-CoViD-19-Patienten, die im selben Zeitraum auf den Intensivstationen des Universitätsklinikums Augsburg behandelt wurden.

Neben der Inzidenz soll geklärt werden, inwiefern das Vorliegen einer TVT auch bei CoViD-19-erkrankten Intensivpatienten mit einer erhöhten Letalität einhergeht. Zudem wird die vorbestehende Antikoagulation zum Zeitpunkt des Auftretens der TVT beschrieben und diskutiert.

## Material und Methoden

Untersucht wurden alle im Zeitraum vom 18.04.2020 bis 30.04.2020 auf der Intensivstation des Universitätsklinikums Augsburg behandelten Patienten mit positivem SARS-CoV2-Nachweis; dabei konnte das Aufnahmedatum bereits vor Studienbeginn liegen. Als Kontrollgruppe diente ein Kollektiv von der internistischen Intensivstation mit Non-CoViD-Patienten im gleichen Zeitfenster. Es gab keine Ausschlusskriterien. Die prospektive Datenerfassung erfolgte mittels Microsoft-Excel® (Version 2010; Microsoft Corporation, Redmond, WA, USA). Insgesamt konnten jeweils 20 intensivpflichtige Patienten mit und 20 ohne positiven PCR-Nachweis auf SARS-CoV2 eingeschlossen werden. Zur Überprüfung der Signifikanz des Vorkommens von TVT bei CoViD-19-Patienten sowie der damit assoziierten Letalität wurde der exakte Test nach Fisher angewandt.

Der Virusnachweiserfolgte durch PCR (Cobas 6800 Kib Roche bzw. Rotor-Gene Kit Altona). Erfasst wurden demographische Daten wie in Tab. 1 dargestellt. Zudem wurden folgende Laborparameter erhoben: Leukozyten, C‑reaktives Protein (CRP), Thrombozyten, Hämoglobin, Kreatinin, INR und D‑Dimer. Zur Erfassung einer disseminierten intravasalen Gerinnung (DIC) wurden die modifizierten Richtlinien der Internationalen Gesellschaft für Thrombose und Hämostase (ISTH) angewandt [13].

**Table 1:** 

	Gesamt		CoVid-19-Patienten		Non-CoVid-19-Patienten	
	(*n*)	(%)	(*n*)	(%)	(*n*)	(%)
*Einbezogene Patienten*	*40*	100,0	*20*	50	*20*	50
Geschlecht (m/w)	27/13		14/6		13/7	
Alter (Jahre) MW ± SD	63,4 ± 18,1		63,7 ± 14,3		63,1 ± 21,5	
Medianes Alter (Jahre)	67		62		69	
BMI (kg/m^2^)	27,8		28,1 ± 4,3		27,4 ± 8,2	
Krankenhausaufenthalt ± SD (Tage)	19,6 ± 12,6		24,4 ± 10,0		14,9 ± 13,3	
Aufenthalt ITS ± SD (Tage)	17,5 ± 13,8		21,5 ± 13,1		13,6 ± 13,7	
TVT	5	12,5	4	10	1	2,5
*Diagnosen*						
CoViD-19-Pneumonie	*20*	50,0	*20*	100	*–*	–
Sepsis	*2*	5,0	*–*	–	*2*	10
Aspirationspneumonie	*1*	2,5	*–*	–	*1*	5
Intoxikation	*1*	2,5	*–*	–	*1*	5
Myokardinfarkt	*3*	7,5	*–*	–	*3*	15
AKI	*1*	2,5	*–*	–	*1*	5
COPD	*2*	5,0	*–*	–	*2*	10
Kardiale Dekompensation	*3*	7,5	*–*	–	*3*	15
Schlaganfall	*1*	2,5	*–*	–	*1*	5
Andere	*6*	15,0	*–*	–	*6*	30
*Vorerkrankungen*						
aHT	*27*	67,5	*13*	65	*14*	70
Diabetes	*12*	30,0	*2*	10	*10*	50
KHK	*9*	22,5	*6*	30	*3*	15
Hyperlipoproteinämie	*12*	30,0	*4*	20	*8*	40
TVT in Anamnese	*2*	5,0	*2*	10	*0*	0
LE in Anamnese	*1*	2,5	*1*	5	*0*	0
*Niktoinabusus*						
Exnikotinabusus	*4*	10,0	*–*	–	*4*	20
Nikotinabusus	*7*	17,5	*2*	10	*5*	25

*aHT* arterielle Hypertonie, *AKI* „acute kidney injury“, *BMI* Body-Mass-Index,* COPD* „chronic obstructive pulmonary disease“, *ITS* Intensivstation, *KHK* koronare Herzkrankheit, *LE* Lungenembolie, *SD* Standardabweichung, *TVT* tiefe Beinvenenthrombose

Die Krankheitsverläufe wurden anhand folgender Daten beurteilt: Entwicklung und Monitoring thrombembolischer Ereignisse, Erfassung und Änderung der gerinnungshemmenden Medikation, Auftreten von nicht-CoViD-assoziierten Erkrankungen, Beatmungstage, Outcome und Letalität.

Bei allen Patienten wurde durch denselben ultraschallerfahrenen Gefäßchirurgen eine Kompressionssonographie der oberflächlichen und tiefen Beinvenen zur Thrombosedetektion im Rahmen eines aktiven Screenings durchgeführt.

Die Patienten wurden entweder bis zur Entlassung von der Intensivstation, bis zu ihrem Tod oder bis zum Studienende am 30.04.2020 beobachtet; je nachdem welches Ereignis zuerst eintrat. Die Studie wurde von der Ethikkommission des Universitätsklinikums Augsburg (Antrags-Nr. 2020-17) genehmigt.

## Ergebnisse

Die Laborparameter zum Zeitpunkt der Ultraschalluntersuchung, das Vorliegen einer TVT, die gerinnungshemmende Medikation, das Vorliegen einer Lungenembolie, die Tage der invasiven Beatmung sowie Letalität der jeweiligen Gruppe sind in Tab. 2 erfasst.

**Table 2:** 

	Gesamt		CoViD-19-Patienten				Non-CoViD-19-Patienten			
			TVT		Non-TVT		TVT		Non-TVT	
	(*n*)	(%)	(*n*)	(%)	(*n*)	(%)	(*n*)	(%)	(*n*)	(%)
*Laborparameter zum Zeitpunkt der Ultraschalluntersuchung*	*40*	100	*4*	20	*16*	80	*1*	5	*19*	95
Leukozyten (/nl) MW ± SD	12,7 ± 8,5		16,0 ± 12,0		10,2 ± 5,1		12,8		14,2 10,1	
CRP (mg/dl) MW ± SD	8,0 ± 8,33		10,8 ± 12,2		9,5 ± 9,7		2,2		6,5 6,3	
Thrombozyten (/nl) MW ± SD	280,8 ± 158,3		289,8 ± 76,2		305,6 ± 126,6		387		250,5 194,8	
Hämoglobin (g/l) MW ± SD	102,7 ± 22,5		93,3 ± 12,0		102,6 ± 16,7		143		102,7	
Kreatinin (mg/dl) MW ± SD	1,2 ± 0,9		1,6 ± 1,3		1,2 ± 0,6		0,54		1,3	
INR MW ± SD	1,1 ± 0,2		1,0 ± 0,2		1,1 ± 0,1		1,1		1,1	
D‑Dimer (ng/ml) MW ± SD	6000,1 ± 6637,6		12.500,8 ± 3840,5		4374,9 ± 6226,6		Nicht erhoben		Nicht erhoben	
*NMH Dosierung bei Aufnahme*										
Prophylaktisch	*9*	22,5	*–*	–	*6*	15,0	*–*	–	*3*	15,0
Halbtherapeutisch	*3*	7,5	*1*	5,0	*2*	5,0	*–*	–	*–*	–
Therapeutisch	*5*	12,5	*2*	10,0	*1*	2,5	*1*	5,0	*1*	5,0
*Unfraktioniertes Heparin*	*17*	42,5	*1*	5,0	*6*	15,0	*–*	–	*10*	50,0
PTT (s) unter Heparin MW ± SD	45,4 ± 18,9		51		42,1 ± 17,1		–		34,3 ± 10,6	
*Noch bestehende Antikoagulation bei Bridging/Switching*	*6*	15	*0*	0	*1*	2,5	*–*	–	*5*	12,5
ASS	*11*	27,5	*–*	–	*4*	10,0	*–*	–	*7*	35,0
Clopidogrel	*2*	5,0	*–*	–	*–*	–	*–*	–	*2*	10,0
Phenprocoumon	*1*	2,5	*–*	–	*–*	–	*–*	–	*1*	5,0
DOAK	*1*	2,5	*–*	–	*–*	–	*–*	–	*1*	5,0
Lungenembolie	*1*	2,5	*1*	25,0	*–*	–	*–*	–	*–*	–
Invasive Beatmung	*20*	50,0	*4*	100,0	*7*	43,8	*1*	100,0	*8*	42,1
*Verstorben im Krankenhaus*	*7*	17,5	*2*	50,0	*3*	18,8	*–*	–	*2*	10,5

*ASS Acetylsalicylsäure, CRP* C-reaktives Protein, *DOAK* direkte orale Antikoagulanzien, *INR* International Normalized Ratio,* NMH* niedermolekulares Heparin. *PTT* partielle Thromboplastinzeit, *SD* Standardabweichung, *TVT* tiefe Beinvenenthrombose

Im Gesamtkollektiv fanden sich 5 TVT: davon traten 4 Thrombosen bei den Patienten mit SARS-CoV2-Nachweis auf (4/20 = 20 %). Bei 3 Patienten im Gesamtkollektiv, respektive 2 in der CoViD-Gruppe, konnte im Rahmen des durchgeführten Screenings eine TVT als Zufallsbefund erstdiagnostiziert werden. Im CoViD-Kollektiv (*n* = 4) traten 3 Thrombosen einseitig auf, nur eine TVT betraf beide Beine. Die Durchführung des exakten Tests nach Fisher zeigte keinen signifikanten Unterschied bezüglich der Inzidenz von TVT zwischen den untersuchten Patientenkollektiven.

Die Altersverteilung bezogen auf die TVT ist in Tab. 3 aufgeführt. Alle CoViD-Patienten mit TVT sind dabei in der Altersgruppe 60 bis 69 Jahre zu finden.

**Table 3:** 

	Gesamt		CoViD-19-Patienten				Non-CoViD-19-Patienten			
			TVT		Non-TVT		TVT		Non-TVT	
	(*n*)	(%)	(*n*)	(%)	(*n*)	(%)	(*n*)	(%)	(*n*)	(%)
*Alter (Jahre)*	40	*100*	4	*20*	16	*80*	1	*5*	19	*95*
<39	*5*	12,5	*0*	0	*1*	5	*1*	5	*3*	15
40–49	*3*	7,5	*0*	0	*3*	15	*0*	0	*0*	0
50–59	*5*	12,5	*0*	0	*3*	15	*0*	0	*2*	10
60–69	*10*	25	*4*	20	*2*	10	*0*	0	*4*	20
>70	*17*	42,5	*0*	0	*7*	35	*0*	0	*10*	50

*TVT* tiefe Beinvenenthrombose

**Figure 1:**
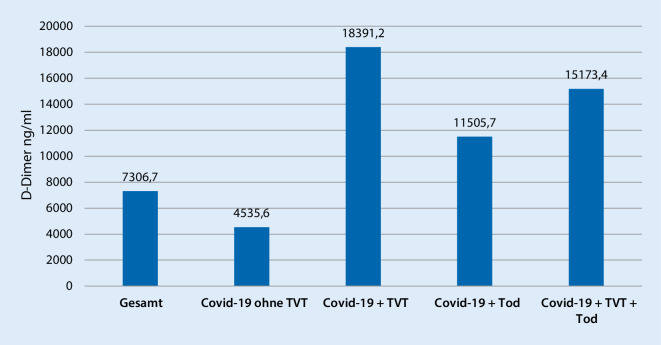

Alle Patienten erhielten ab Aufnahmezeitpunkt eine Thromboseprophylaxe mit NMH s.c. oder befanden sich noch im therapeutischen Bereich bei vorbestehender Vollantikoagulation mittels DOAK. Zum Zeitpunkt der Kompressionssonographie waren 3 der 4 CoViD-TVT-Patienten bereits therapeutisch antikoaguliert: Davon 2 bei bereits bekannter, neu aufgetretener TVT und ein Patient der sich im Bridging bei Vorhofflimmern befand. Bei dem 4. Patienten mit NMH in halbtherapeutischer Dosierung (Enoxaprin 0,4 s.c. 1–0–1) bei erhöhtem Thromboserisiko bei Adipositas und tachykarder Herzrhythmusstörung erfolgte ein Wechsel des medikamentösen Regimes hin zur volltherapeutischen Antikoagulation. Bei einem der CoViD-Patienten trat eine Progression der Thrombose sowie eine fulminante Lungenarterienembolie (LAE) auf, welche zum Tode führte. In der Kontrollgruppe zeigte sich nur ein Fall von komplikationsloser TVT. Bei der Diagnosestellung der Thrombose lag bei keinem Patienten eine DIC vor. Der an der LAE verstorbene Patient entwickelte erst im späten Krankheitsverlauf eine DIC. Abb. 1 zeigt die Mittelwerte der D‑Dimer-Bestimmung im stationären Verlauf in den einzelnen Gruppen.

Von den mit SARS-CoV2 infizierten Patienten sind 5 (25 %), in der Kontrollgruppe 2 Patienten (10 %) verstorben. Tab. 4 stellt das Outcome bezüglich Tod, Verlegung und Entlassung dar. Ursächlich für den Tod der 5 CoViD-Patienten waren in einem Fall eine Lungenembolie, in einem weiteren Fall Multiorganversagen bei septischem Schock sowie dreimal ein „acute respiratory distress syndrom“ (ARDS) bei CoViD-19-Pneumonie. Unter den Verstorbenen waren 2 Patienten mit TVT (Todesursache ARDS bzw. LAE). Insgesamt zeigte sich im Kollektiv der CoViD-19-Patienten mit TVT eine Letalität von 50 % verglichen mit 19 % bei den CoViD-19-Patienten ohne TVT. Die Durchführung des exakten Tests nach Fisher zeigte auch hier keinen signifikanten Unterschied.

**Table 4:** 

	Gesamt		CoViD-19-Patienten		Non-CoViD-19-Patienten	
	(*n*)	(%)	(*n*)	(%)	(*n*)	(%)
Bei Studienende noch im KH	*28*	70,0	*14*	70	*14*	70
Entlassen	*5*	12,5	*1*	5	*4*	20
Tod	*7*	17,5	*5*	25	*2*	10

*KH* Krankenhaus

## Diskussion

Vorangegangene Publikationen beschreiben die Rate an tiefen Beinvenenthrombosen bei intensivpflichtigen CoViD-19-Patienten mit 1–56 % [1, 5, 7]. Im aktuellen Kollektiv zeigte sich eine TVT-Rate von 20 % bei den CoViD-19-Patienten, verglichen mit 5 % bei den Non-CoViD-19-Patienten der Kontrollgruppe. Die Inzidenz einer TVT bei vorliegendem SARS-CoV2-Nachweis zeigte sich damit zwar nicht signifikant, jedoch deutlich erhöht. Das untersuchte Kollektiv weist insgesamt nur eine limitierte Patientenzahl auf, was Rückschlüsse auf die tatsächliche Inzidenz der TVT bei CoViD-19-Patienten erschwert. Zudem befand sich ein Großteil der Patienten (70 %) nach Studienende noch in stationärer Behandlung, wodurch grundsätzlich das Auftreten weiterer TVT und damit ein Anstieg der Inzidenz denkbar ist. Dem gegenüber steht die Tatsache, dass die Patienten der vorliegenden Studie aktiv auf eine TVT gescreent wurden. Insgesamt sind die Ergebnisse bezüglich der gesteigerten Inzidenz von TVT in der vorliegenden Studie bei SARS-CoV2-positiven Intensivpatienten mit der aktuellen Datenlage vergleichbar [1, 5, 7].

Wie das SARS-CoV2-Virus dabei konkret Einfluss auf das Gerinnungssystem und damit assoziierte thrombembolische Ereignisse nimmt, konnte in der vorliegenden Arbeit nicht eruiert werden. Prinzipiell gibt es hierzu zwei unterschiedliche Thesen: Einerseits könnte das SARS-CoV2-Virus die Gefäßwand, getriggert durch immunologische Prozesse, direkt angreifen und somit zu einer lokalen Thrombose, ausgelöst durch einen Intimaschaden, führen [6, 14]. So konnten Varga et al. post mortem Viruspartikel in Endothelzellen von CoViD-19-Patienten nachweisen [15]. Hierzu passt die von Klok et al. publizierte niedrige Rate von 1 % TVT bei einem Patientenkollektiv von 184 intensivpflichtigen CoViD-19-Patienten – unabhängig von der niedrigen Rate an TVT hatte sich bei 14 % der Patienten eine Lungenarterienembolie gezeigt, was das Konzept einer lokalen Thrombose (und nicht einer konventionellen Thrombembolie) stützt [5].

Die Ergebnisse der aktuellen Studie deuten jedoch noch in eine andere Richtung: Lediglich ein Patient erlitt eine Lungenarterienembolie, bot aber auch gleichzeitig die Diagnose einer TVT, was zum Bild der klassischen Thrombembolie passt. Insofern erscheint ein anderer Aspekt nicht minder wichtig: Die CoViD-19-Infektion führt nicht nur zu einem lokalen, sondern vielmehr auch zu einem systemischen Geschehen im Sinne einer vermutlich immunologisch getriggerten Koagulopathie. Dieser Ansatz wird von nahezu allen bisher publizierten Arbeiten zu thrombembolischen Ereignissen bei intensivpflichtigen CoViD-19-Patienten postuliert und erklärt die deutlich erhöhte Anzahl von TVT [1–3, 7, 16].

Interessanterweise entwickelte ein CoViD-19-Patient trotz vorbestehender Antikoagulation eine TVT. Obwohl sich diese Tatsache einer einfachen Erklärung entzieht, so spiegelt sie dennoch die Ergebnisse von Llitjos et al. wider: Hier kam es bei 56 % der bereits therapeutisch antikoagulierten Patienten zu einer TVT, von denen wiederum über die Hälfte (60 %) mit einer Lungenarterienembolie vergesellschaftet waren [7].

Im vorliegenden Kollektiv der CoViD-19-Patienten mit TVT zeigte sich zudem eine Letalität von 50 % verglichen mit 19 % bei den Patienten ohne TVT. Auch wenn dieser Unterschied nicht signifikant war, so deckt sich dieses Ergebnis mit der von Cui et al. publizierten Studie, die für SARS-CoV2-positive intensivpflichtige Patienten mit TVT eine Letalität von 32 % aufzeigen konnte, und zeigt eindrücklich die potenzielle Relevanz einer TVT als Prognosefaktor für eine erhöhte Letalität. Einen ähnlichen prognostischen Wert scheinen die D‑Dimere zu haben: Die höchsten D‑Dimere in der aktuellen Studie zeigten sich bei den CoViD-19-Patienten, welche im Verlauf eine TVT entwickelten und/oder einen tödlichen Verlauf nahmen. Diese Ergebnisse stützen die Untersuchungen von Tang et al., die für SARS-CoV2-positive intensivpflichtige Patienten eine schlechtere Prognose quo ad vitam beim Vorliegen erhöhter D‑Dimere feststellen konnten [17].

Umso wichtiger erscheint unter diesen Aspekten die frühzeitige Diagnosestellung einer Koagulopathie und der damit assoziierten thrombembolischen Ereignisse. In Anlehnung an die bereits publizierte Konsensusempfehlung der Internationalen Gesellschaft für Thrombose und Hämostase (ISTH) sowie aufgrund der aktuellen Studienlage halten wir die standardmäßige D‑Dimer-Erhebung zum Aufnahmezeitpunkt bei stationären und insbesondere intensivpflichtigen SARS-CoV2-positiven Patienten für sinnvoll [18]. Zudem sollte bei erhöhten D‑Dimeren und/oder klinischen Thrombosezeichen eine Kompressionssonographie des tiefen Venensystems erfolgen, um die generell empfohlene Antikoagulation mit niedermolekularem Heparin in prophylaktischer Dosierung gegebenenfalls therapeutisch anpassen zu können [16, 19].

Ein Nachteil der Bestimmung von D‑Dimeren ist jedoch die Tatsache, dass diese zwar Hinweise auf eine bereits vorliegende Thrombose geben, nicht aber schon vorher eine Hyperkoagulabilität detektieren können. Zielführender wären insofern Tests, die eine Hyperkoagulabilität feststellen, bevor es zur Ausbildung einer TVT kommt, und so ggf. eine präventive Antikoagulation ermöglichen würden. Hilfestellung könnten hier Point-of-care-Methoden bieten. So berichtete Panigada et al. von der Thromboelastographie (TEG®; Haemonetics TEG 5000, Braintree, MA), welche eine schnell verfügbare Aussage zu den viskoelastischen Eigenschaften von Blut erlaubt. Auch hier zeigte sich, dass CoViD-19-Patienten eine erhöhte Koagulabilität mit dem damit assoziierten Risiko für thrombembolische Ereignisse aufweisen [20]. Eine weitere Point-of-care-Methode stellt das Rotations-Thromb-Elastogramm (ROTEM®, Tem Innovations GmbH, München) dar: Ranucci et al. konnten bei 16 intensivpflichtigen SARS-CoV2-positiven Patienten mit ARDS mittels ROTEM ebenfalls eine erhöhte Koagulabilität nachweisen [21]. Um den Stellenwert dieser Methoden im Rahmen einer CoViD-19-Infektion endgültig beurteilen zu können, sind jedoch weitere Studien notwendig. Bis dahin erscheinen die o. g. Konsensusempfehlungen der ISTH sinnvoll.

## Fazit für die Praxis

- Die CoViD-19-Infektion ist mit einem erhöhten Risiko für thrombembolische Ereignisse vergesellschaftet.
- Die Rate an tiefen Beinvenenthrombosen (TVT) war im untersuchten Kollektiv bei Patienten mit SARS-CoV2 deutlich erhöht (CoViD-19-Patienten: 20 % vs. Non-CoViD-19-Patienten: 5 %).
- Sowohl das Vorliegen einer TVT sowie deutlich erhöhte D‑Dimer-Werte waren in der vorliegenden Studie mit erhöhter Letalität vergesellschaftet.
- Wir empfehlen bei der stationären Aufnahme von Patienten mit SARS-CoV2-Verdacht oder -Nachweis die Bestimmung der D‑Dimere und im Falle erhöhter Werte die großzügige Indikationsstellung zur Kompressionssonographie der tiefen Beinvenen. So können TVT früh erkannt und eine therapeutische Antikoagulation begonnen werden.
- Alle stationären CoViD-19-Patienten sollten eine Thromboseprophylaxe mit niedermolekularem Heparin erhalten.
- Weitere Studien zu Point-of-care-Methoden (TEG®, ROTEM®) zur Erkennung einer Hyperkoagulabilität bei SARS-CoV2 sind notwendig.
